# Huge Cystic Lymphangioma of the Pancreas Mimicking Pancreatic Cystic Neoplasm

**DOI:** 10.1155/2012/951358

**Published:** 2012-11-06

**Authors:** Enrico Dalla Bona, Valentina Beltrame, Stella Blandamura, Federica Liessi, Cosimo Sperti

**Affiliations:** ^1^Department of Surgery, Oncology, and Gastroenterology, University of Padua, 35128 Padua, Italy; ^2^Department of Pathology, University of Padua, 35128 Padua, Italy; ^3^Department of Radiology, Bassano General Hospital, 36061 Bassano, Vicenza, Italy

## Abstract

Lymphangiomas of the pancreas are very rare benign tumors of lymphatic origin, accounting for less than 1% of these neoplasms. We report a case of a 55-year-old woman who presented with a palpable mass in the left abdomen. Abdominal sonography and computed tomography showed a lobulated, hypodense mass extending from the left diaphragm to the pelvis, measuring 10 × 25 cm. A preoperative diagnosis of mucinous cystadenoma of the pancreas was suggested and the patient underwent laparotomy. Distal pancreatectomy with splenectomy was performed, encompassing a segment of descending colon because of close relationship to the mass. The cystic mass was histologically diagnosed as lymphangioma of the pancreas. The patient is well and free of disease 12 months after surgery. Pancreatic lymphangioma should be kept in mind when a huge, multiloculated mass is encountered in the abdomen, especially in adult women. Although lymphangioma is considered a benign tumor, involvement of adjacent organs sometimes occurs and extended resection is required to obtain a radical treatment.

## 1. Introduction

Lymphangiomas are rare, benign lesions which originate from lymphatic vessels and occur most frequently in children [1, 2]. Lymphangiomas typically arise in the head, neck (75%), and axilla region (20%), but they are described in other sites, like mediastinum, pleura, pericardium, groin, bones, and the abdomen [3]. The pancreas is a very rare localization of lymphangioma (1% of these tumors) [1, 4] that represents only 0.2% of pancreatic neoplasms [5]. Since the first description made by Koch in 1913 [6], there were only about 70 reported cases of pancreatic lymphangiomas in a recent review of the literature made by Ghatak et al. in 2011 [7]. We report a case of a 55-year-old woman with a huge cystic lymphangioma of the tail of the pancreas, mimicking a pancreatic cystic neoplasm, and treated with extended surgical resection.

## 2. Case Report

**Figure 1:**
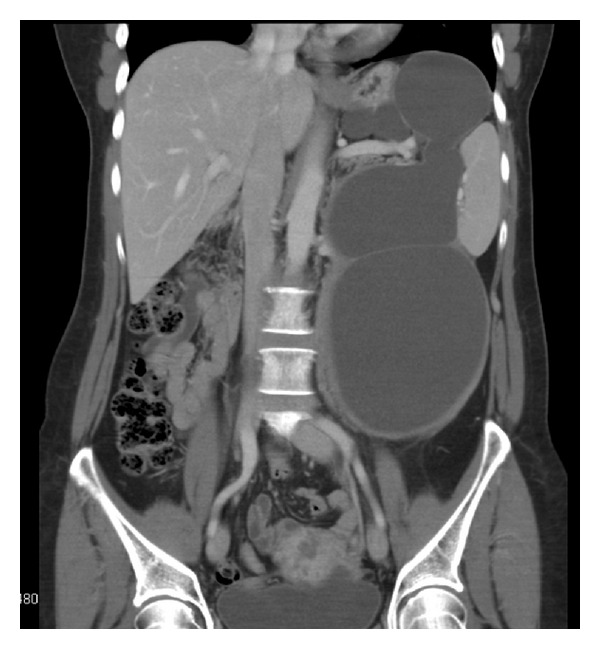
CT scan of the abdomen (coronal view) showing a large cystic mass extending from the diaphragm to the left iliac region.

**Figure 2:**
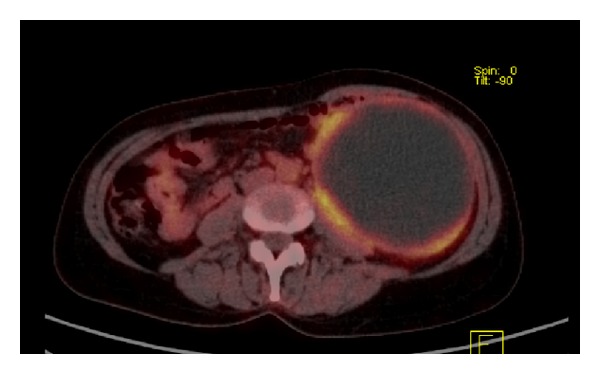
PET/CT scan of the abdomen (sagittal view) showing a cystic mass with peripheral uptake of 18-FDG.

A 55-year-old female presented with a one-month history of moderate pain on the left side of the abdomen. Physical examination revealed a huge, palpable mass occupying the left region of the abdomen. Blood tests, including tumor markers carcinoembryonic antigen (CEA), carbohydrate antigens CA 19-9, CA 125, and CA 15-3 were within the normal limit. Ultrasound of the abdomen showed a large, hypoechoic mass with internal septae, of about 17 cm of diameter. Abdominal computed tomography (CT) showed that the mass (measuring 10 × 25 cm, with fluid content) was extended in the left side of the abdomen from the diaphragm to the left iliac region, adherent to the tail of the pancreas and the splenic vessels (Figure 1). 18-FDG-positron emission tomography with CT acquisition (PET/CT) showed a peripheral, low uptake of the radiotracer, with no sign of metabolic activity into the mass (Figure 2). Preoperative suspicion of mucinous cystic neoplasm of the pancreas was made and surgery was planned. 

**Figure 3:**
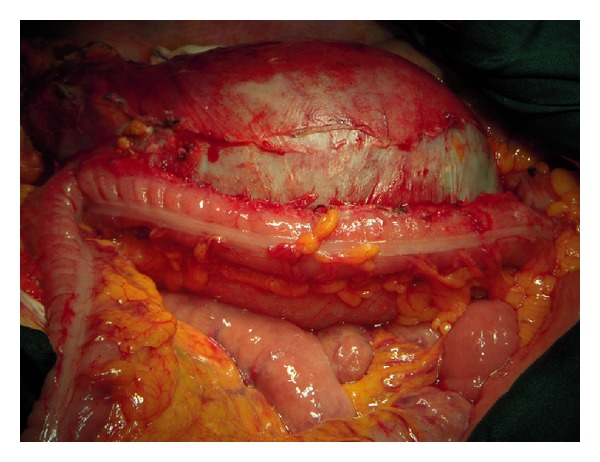
Intraoperative finding—the cystic mass involving the descending mesocolon.

**Figure 4:**
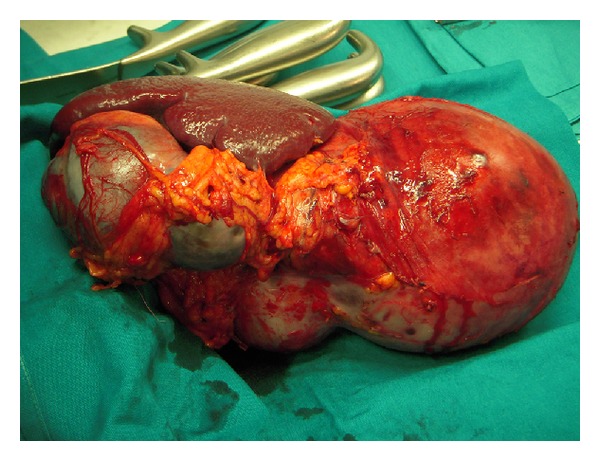
Intraoperative finding—en bloc resection of the mass with the tail of the pancreas and the spleen.

**Figure 5:**
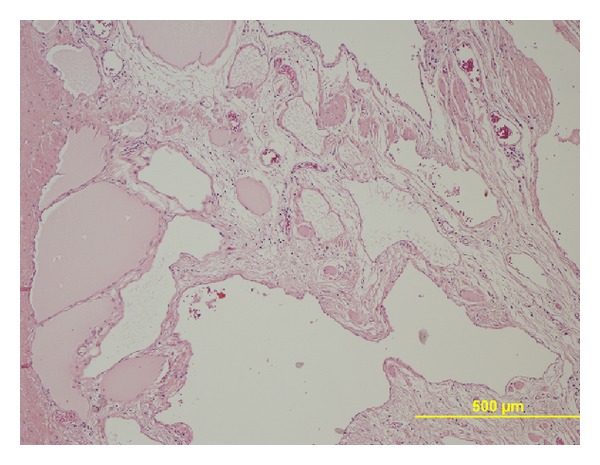
Histological sample—ectatic lymphatic vessels inside the mass (H & E, 100x).

At laparotomy, in September 2011, the left peritoneal cavity was occupied by a huge mass, with thick capsule, arising from the tail of the pancreas, and extending from the diaphragm to the left iliac region. The lesion infiltrated the spleen and the descending colon was tightly adherent to it for at least 20 cm (Figure 3). To perform “en bloc” radical resection of the mass, distal pancreatectomy with splenectomy (Figure 4) and excision of 30 cm of descending colon were required: reconstruction of the colon was performed with end to end anastomosis. Microscopically, the specimen showed enlarged cystic-like spaces, lined by endothelial cells contained within lymph-like fluid (Figure 5). Resection margins of the pancreas and colon were free from neoplastic infiltration. The endothelial cells lining the surface of the cystic space were positive for CD31 and D2-40; so, the diagnosis of lymphangioma was made. The postoperative course was uneventful and the patient was discharged on the 7th day after the operation. The patient is well and free of tumor's recurrence 12 months after surgery.

## 3. Discussion

Lymphangiomas are considered rare benign tumors. The etiology of lymphangioma remains unclear; a well-established theory suggests that they develop from a congenital malformation of lymphatic vessels, leading to blockage of lymphatic flow and lymphangiectasia [8]. Other authors suggest that these tumors are the consequence of inflammation, leading to obstruction in lymphatic channels [9]. Histologically there are 3 types of lymphangiomas: capillary, cavernous, and cystic [10]. Pancreatic lymphangiomas are most commonly found in females; in a literature review by Igarashi et al. [11] including 45 cases of pancreatic lymphangioma, the female : male ratio was 29 : 16 and the age of the patients ranged from 2 to 81 years, with a mean age of 40 years. These tumors may be asymptomatic for a long time; the most common symptoms are abdominal pain and awareness of abdominal mass [9]. Other infrequent symptoms such as vomiting or nausea are caused by occupied tumor. Although rare, acute abdomen can be the first sign of presentation due to complications, such as intestinal obstruction, rupture and/or hemorrhage [9, 12, 13]. Since these tumors are very rare, the cystic aspect can confuse the proper diagnosis. Differential diagnosis includes nonneoplastic cystic lesion (usually unilocular) or Echinococcus cysts (usually with calcifications); neoplastic cystic tumors of the pancreas which have to be considered in differential diagnosis are mucinous [11], macrocystic serous cystadenoma, and branch type IPMN [7, 8, 10]. There are no specific blood markers to confirm the diagnosis of lymphangioma of the pancreas, moreover radiological imaging (US, TC, MRI) often are not able to perform a correct preoperative diagnosis [8, 10]. Usually, the diagnosis of pancreatic lymphangioma is established only after histological examination of the surgical specimen, with endothelial cells showing positivity for CD31 or CD34 or factor VIII/R antigen [8]. Some Authors [14–16] advocate the usefulness of endoscopic ultrasound (EUS) in the preoperative workup of suspected lymphangiomas: the analysis of cyst fluid by fine-needle aspiration (FNA) for cytology and triglycerides determination may help in differential diagnosis. In our patient, we did not perform EUS-FNA since we promptly planned a surgical management because of the large size of the lesion, its symptoms, and the suspect of mucinous cystic tumor of the pancreas. 

The treatment of choice of these lesions remains the complete surgical resection, which provides excellent prognosis and very low incidence of recurrence (about 7%) [17]; partial resection is associated with a high recurrence rate, 50% in one series after a mean follow-up period of two years [18]. Although lymphangioma is a benign tumor, it often behaves in an aggressively manner and can grow to an enormous size. Therefore, resection of adjacent organs may be required to accomplish complete excision, as in our case. Egurney et al. [19] recently reported a similar case of huge lymphangioma of the head of the pancreas inseparable from the transverse mesocolon and the greater curvature of the stomach. Excision of the mass was performed with pancreaticoduodenectomy, transverse colectomy, and distal gastric resection. Malignant transformation to lymphosarcoma or adenocarcinoma have been also reported [18], but are exceedingly rare [20].

## 4. Conclusion

It is still difficult to establish a correct preoperative diagnosis of a pancreatic cystic neoplasm. Although rare, lymphangioma should be included in the differential diagnosis of a complex intra-abdominal cystic mass, especially in poorly symptomatic women.
